# Novel Ionic Conducting Composite Membrane Based on Polymerizable Ionic Liquids

**DOI:** 10.3390/polym13213704

**Published:** 2021-10-27

**Authors:** Yaroslav L. Kobzar, Ghania Azzouz, Hashim Albadri, Jocelyne Levillain, Isabelle Dez, Annie-Claude Gaumont, Laurence Lecamp, Corinne Chappey, Stéphane Marais, Kateryna Fatyeyeva

**Affiliations:** 1 Normandie Université, UNIROUEN, INSA Rouen, CNRS, Polymerès Biopolymères Surfaces (PBS), 76000 Rouen, France; yaroslav.kobzar@univ-rouen.fr (Y.L.K.); ghania.azzouz@univ-rouen.fr (G.A.); laurence.lecamp@insa-rouen.fr (L.L.); corinne.chappey@univ-rouen.fr (C.C.); stephane.marais@univ-rouen.fr (S.M.); 2 Normandie Université, ENSICAEN, UNICAEN, CNRS, Laboratoire de Chimie Moléculaire et Thioorganique, 14000 Caen, France; Hashim.Albadri@ensicaen.fr (H.A.); Jocelyne.Levillain@ensicaen.fr (J.L.); isbelle.dez@ensicaen.fr (I.D.); annie-claude.gaumont@ensicaen.fr (A.-C.G.)

**Keywords:** polymerizable ionic liquid, polymer membranes, conductivity, photopolymerization, fuel cell

## Abstract

In this work, the design and characterization of new supported ionic liquid membranes, as medium-temperature polymer electrolyte membranes for fuel-cell application, are described. These membranes were elaborated by the impregnation of porous polyimide Matrimid^®^ with different synthesized protic ionic liquids containing polymerizable vinyl, allyl, or methacrylate groups. The ionic liquid polymerization was optimized in terms of the nature of the used (photo)initiator, its quantity, and reaction duration. The mechanical and thermal properties, as well as the proton conductivities of the supported ionic liquid membranes were analyzed in dynamic and static modes, as a function of the chemical structure of the protic ionic liquid. The obtained membranes were found to be flexible with Young’s modulus and elongation at break values were equal to 1371 MPa and 271%, respectively. Besides, these membranes exhibited high thermal stability with initial decomposition temperatures > 300 °C. In addition, the resulting supported membranes possessed good proton conductivity over a wide temperature range (from 30 to 150 °C). For example, the three-component Matrimid^®^/vinylimidazolium/polyvinylimidazolium trifluoromethane sulfonate membrane showed the highest proton conductivity—~5 × 10^−2^ mS/cm and ~0.1 mS/cm at 100 °C and 150 °C, respectively. This result makes the obtained membranes attractive for medium-temperature fuel-cell application.

## 1. Introduction

The implementation of renewable eco-friendly energy sources, such as solar, wind, and hydropower, over fossil fuels is progressively gaining momentum. However, most of these sources are intermittent, opening spatial and temporal gaps between the availability of the energy and its consumption by end-users [1]. The development of alternative energy technologies without this drawback is one of the ways for meeting the world’s energy needs in the future. Polymer exchange membrane fuel cells (PEMFCs) that can operate at high temperatures (above 100 °C) without additional humidification are promising conductive systems for future energy technology due to their efficiency and eco-friendly nature [2,3]. Such membranes possess several advantages over currently used polymer electrolytes for fuel cells; for example, a reduction in the amount of Pt used, as well as the use of cheaper catalysts, and the use of hydrogen with a relatively high carbon monoxide content are possible at elevated temperatures [3]. Besides these, the heat generated by the fuel cell can be used, and the cell’s temperature control and water management is easier in such cells; thus, the fuel cell’s volume can be reduced [1,4]. However, the development of a new proton-conductive membrane, designed for a long operation period under medium- and high-temperature anhydrous conditions (>100 °C) is a key issue. PEM should meet several requirements; namely, it should have high proton conductivity, negligible electronic conductivity, low permeability to fuel, thermal, hydrolytic and oxidative stability under operating conditions, and good mechanical properties [1,2,3,4].

Up to now, several types of polymers have been tested in medium- and high-temperature PEMFCs, namely polyimides, polysulfones, polybenzoxazoles, poly(ether ether ketone)s, polybenzimidazoles, polyoxadiazoles, and nanostructured organic-inorganic composites [5,6,7,8,9,10,11,12,13]. The H_3_PO_4_ doping of these polymers is one of the easiest and most often-used approaches to design proton-conductive membranes. H_3_PO_4_ exhibits characteristics of both a proton-donor (due to the formation of H_2_PO_4_^−^/H_4_PO_4_^+^ ion pairs) and a proton-conducting medium [14]. The main disadvantages of such membranes are the loss of the membrane’s mechanical stability, the acid release during fuel-cell operation, and acid adsorption onto the surface of the Pt catalyst, which hampers the reaction kinetics [15].

Ionic liquids (ILs) have attracted considerable attention due to their unique properties, such as low vapor pressure, high thermal stability, wide electrochemical window, high ion conductivity, etc. Today, the number of ILs described in the literature is very large [1,16,17]. Most ILs are protic ionic liquids (PILs), which can be easily produced through the combination of a Brønsted acid and a Brønsted base [18]. The key feature of PILs is the presence of proton-donor and proton-acceptor sites, due to the proton transfer from the acid to the base. Owing to this fact, PILs are insensitive to the presence of water and other proton substances, they have a low melting point, low viscosity, and high thermal and chemical stability, which is very important for their use in electrochemical processes [19,20]. This fact distinguishes PILs from other types of ILs and can be used to form a hydrogen-bonded network [18]. All these properties make PILs an attractive alternative to H_3_PO_4_ for next-generation electrolytes in electrochemical devices.

There are various strategies for developing IL-based PEMs: (1) the synthesis of polymer analogues of ILs [21,22], (2) the *in-situ* polymerization of monomers in IL [23], (3) the solution casting of IL/polymer solutions [1], and (4) the swelling of the polymer membrane in IL [16,24,25,26]. However, one of the main limitations of such membranes is the releasing of ILs during fuel cell operation. In order to prevent such release, a combination of both PILs and their polymer analogues can be used, as ionic interactions between the PIL and the polymer ionic liquid can ensure the presence of ionically cross-linked ionomer networks, thus limiting the PILs’ migration. In addition, PIL-based systems will be much cheaper, as compared with acid-based systems, due to the unique properties of PILs. Besides, PILs can also protect metallic components, i.e., the piping and bipolar plates, from corrosion [14,15,16,17,18,19,20]. In the present work different polymerizable ILs were synthesized and characterized in terms of their conductivity value. The porous polyimide Matrimid^®^ matrix, prepared according to the phase inversion technique [25,26], was used as a polymer support. Matrimid^®^ was selected due to its high chemical, thermal (thermal degradation > 450 °C), and mechanical resistance [25,27], and also its excellent solubility [25]. The supported IL membranes were elaborated by the impregnation of the porous Matrimid^®^ membrane in synthesized polymerizable ILs. The IL polymerization reaction was optimized in terms of the nature and content of the (photo)initiator and the reaction duration. The elaborated supported ionic liquid membranes (SILMs) were characterized by different physical–chemical analyses in order to optimize their composition, thermal and electrochemical properties. In addition, the conductivity properties of the membranes were correlated with their structure and composition.

## 2. Experimental Section

### 2.1. Materials

*N*-vinyl imidazole (≥99%), *N*-allyl imidazole (≥90%), *N*-methylpyrrolidone (NMP) (≥99%), polyvinylpyrrolidone (PVP) (M_w_ 360,000, 90% purity) were purchased from Sigma-Aldrich (St. Quentin Fallavier Cedex 38297,France). Trifluoromethanesulfonic acid (>98%) and benzophenone (≥99%) were purchased from Alfa Aesar (France), and Irgacure^®^ 819 was purchased from Ciba Specialty Chemicals (Switzerland). All chemicals were used as received, without further purification. Polyimide Matrimid^®^ 5218 (glass transition temperature *T*_g_ = 323 °C) was kindly provided by Huntsman^®^ Advanced Materials (Switzerland), and 2-(1-imidazolyl)ethyl methacrylate was prepared according to the following procedure [28].

### 2.2. Synthesis of Ionic Liquids

#### 2.2.1. Vinyl Imidazolium Trifluoromethane Sulfonate (VImTf)

**Scheme 1:**
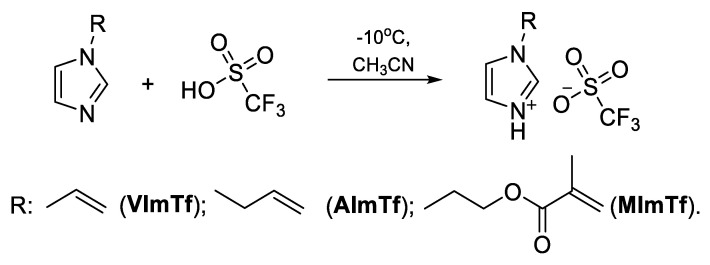
Synthesis of protic imidazolium-based ionic liquids—VImTf, AImTf and MImTf.

*N*-vinyl imidazole (9.4 g, 1.0 mmol) was dissolved in 200 mL of dry acetonitrile at −10 °C under the argon atmosphere (Scheme 1). Then, the trifluoromethanesulfonic acid (15.0 g, 1.0 mmol) was slowly added, one drop at a time, to the solution, under stirring. During the mixing, the temperature of the solution did not exceed −5 °C. The reaction mixture was stirred for additional 2 h at 20 °C, and then the solvent was evaporated under vacuum. The obtained salt was washed twice by ether solvent (100 mL), filtered, and dried in a vacuum oven at 80 °C for 24 h.

Melting temperature *T*_m_ = 77 °C (by DSC). ^1^H NMR (DMSO-*d_6_*, 400 MHz, δ, ppm): 9.34 (s, 1H), 8.16 (s, 1H), 7.79 (s, 1H), 7.28 (m, 1H), 5.99–5.93 (dd, 1H), 5.42–5.39 (dd, 1H). ^19^F NMR (DMSO-*d_6_*, 400 MHz, δ, ppm): −77.82 (s, 3F, -CF_3_).

#### 2.2.2. Allyl Imidazolium Trifluoromethane Sulfonate (AImTf)

AImTf was prepared in the same manner as VImTf with the use of *N*-allyl imidazole instead of *N*-vinyl imidazole (Scheme 1).

*T*_m_ = 11.7 °C (by DSC). ^1^H NMR (MeOD, 500 MHz, δ, ppm): 8.95 (s, 1H), 7.63–7.60 (d, 2H), 6.11 (m, 1H), 5.46–5.41 (m, 2H), 4.92 (d, 2H). ^19^F NMR (MeOD, 400 MHz, δ, ppm): −80.10 (s, 3F, -CF_3_).

#### 2.2.3. Methacrylate Imidazolium Trifluoromethane Sulfonate (MImTf)

MImTf was prepared in the same manner as VImTf using methacrylate imidazole instead of *N*-vinyl imidazole (Scheme 1).

^1^H NMR (DMSO-*d_6_*, 500 MHz, δ, ppm): 9.01 (m, 2H), 7.79 (s, 1H), 7.67 (s, 1H), 6.01 (s, 1H), 5.71 (s, 1H), 4.48 (d, 2H), 3.74 (d, 2H), 1.83 (s, 3H). ^19^F NMR (DMSO-*d_6_*, 400 MHz, δ, ppm): −79.63 (s, 3F, -CF_3_).

### 2.3. Ionic Liquid Photopolymerization

The polymerization of VImTf was performed in the presence of Irgacure^®^ 819. For this purpose Irgacure^®^ 819 photoinitiator (1 wt.%) was mixed with VImTf (1.0 g, 5.6 mmol) at 25 °C. Then, the mixture was heated up to 80 °C until VImTf was melted for better mixing. The polymerization was carried out by the irradiation of polychromatic radiation using a Hg-Xe lamp (Hamamatsu LC8). The light intensity was measured at the sample surface using a radiometer (Vilber-Intraspec Oriel VLX-3W) and set to 165 mW/cm^2^ at λ = 365 nm. The photopolymerization reaction was followed by real-time infrared spectroscopy using a Vertex 70 FTIR spectrometer (Bruker). An attenuated total reflection accessory with controlled temperature (ATR MKII Golden Gate, SPecac, Eurolabo) was used. The resulting polymer, PVImTf, in the form of a white solid, was dissolved in methanol and precipitated into an excess of acetone. The resulting white precipitate was filtered and dried for 1 day in an oven at 60 °C.

### 2.4. Preparation of Porous Polyimide Matrimid^®^ Membrane

The porous membrane was prepared by a vapour-induced phase-separation process, according to the procedure described in [25]. Briefly, Matrimid^®^ (14 wt.%) was dissolved in NMP with PVP (7 wt.%) and the solution was heated at 70 °C and stirred for about 6 h in a closed flask until a homogeneous mixture was obtained. The resulting viscous solution was cast with a Doctor Blade on a glass plate and then exposed to a humid nitrogen stream at 25 °C and 50% of relative humidity for 6 h. After formation, the membrane (with a final thickness of 100 μm) was peeled off in pure water and washed thoroughly in a water/ethanol mixture (50/50 vol.%) to eliminate PVP and NMP. The porous support was dried in the ambient air and finally stored in a desiccator under vacuum.

### 2.5. Impregnation of Porous Matrimid^®^ Membrane with Ionic Liquids

The incorporation of PILs in the porous Matrimid^®^ membrane was carried out by impregnation using the direct immersion method. Previously, it was found that 5 h of impregnation was sufficient for this kind of membrane [24]. In our case, in order to ensure that the maximum mass uptake was reached and due to the high viscosity of IL, the impregnation time was about 12 h. The membranes before impregnation were dried at 80 °C in a vacuum for 24 h. The impregnation step was performed at 25 °C for AImTf and at 80 °C for VImTf. After impregnation, the membranes were delicately wiped to remove excess PIL from the membrane surface.

The impregnation of the polymer PIL (i.e., PVImTf) was performed in the water solution of PVImTf (66.7%) in several steps. First, the Matrimid^®^ membrane was immersed in the solution containing PVImTf and left there for 2 h at 25 °C. Then, the membrane was dried in the air for 3 h. This procedure was repeated twice. After that, the membrane was again immersed in the PVImTf solution at 40 °C for 2.5 h. Finally, the membrane was taken from the solution and gently scraped to remove the excess of PIL from its surface, left in the air for 2 h and vacuum dried at 80 °C during 24 h.

To determine the immobilized PIL quantity in the membrane *τ*, all membranes were weighted before *m_dry_* and after *m_impregnated_* their impregnation:

(1) $$\tau \left(\%\right)=\frac{m_{impregnated}-\;m_{dry}\;}{m_{dry}}\;\cdot 100.$$

For further analysis, all prepared SILMs were stored under vacuum and dried at 80 °C just before characterization.

### 2.6. Characterization Techniques

#### 2.6.1. Fourier Transform Infrared (FTIR) Spectroscopy

FTIR spectra of the synthesized PILs and obtained during the IL thermally initiated polymerization reaction were measured using an Avatar 360 FTIR spectrometer (Thermo Fisher, Waltham, MA, USA) in the attenuated total reflectance (ATR) mode using a Ge crystal. The spectra were the results of 64 coadded interferograms at a 4 cm^−1^ resolution between 600 and 4000 cm^−1^.

#### 2.6.2. Differential Scanning Calorimetry (DSC)

DSC measurements were performed by Q2000 DSC (TA Instruments, New Castle, DE, USA) calorimeter under the nitrogen atmosphere. For that purpose, the sample weighing 4–6 mg was sealed in the aluminum pan. The test procedure included two cycles as a heating–cooling–heating protocol, which started with the sample cooling from the ambient temperature to −80 °C and the temperature rising up to 140 °C (in case of MImTf), 200 °C (in case of AImTf) or 250 °C (in case of VImTf). Then, the temperature was kept constant for 1 min to erase its thermal history. Next, cooling was initiated to reach −80 °C. Finally, the second heating cycle was carried out. The heating/cooling rate throughout the whole cycles was at 10 °C/min.

#### 2.6.3. Thermogravimetric Analysis (TGA)

TGA curves were obtained using Q500 TGA (TA Instruments, New Castle, DE, USA) with flowing nitrogen in dynamic and static modes. The heating rate of 20 °C/min within the temperature range from 25 °C to 800 °C was applied in a dynamic mode. In a static TGA mode, the PILs were maintained at a constant temperature (i.e., at 130 °C) during 900 min and the mass change was recorded.

#### 2.6.4. Viscosity Measurements

An Advanced Rheometer parallel plate rheometer (AR1000, TA Instruments) was used to study the PIL viscosity *η* at 25 °C and 80 °C. The geometry used was a 6-cm diameter steel parallel plate, and the tests were performed in a flow mode using a sample gap of 0.8 mm. All measurements were conducted at the atmospheric pressure.

#### 2.6.5. Scanning Electron Microscopy (SEM) and Optical Microscopy

Microstructural analysis of the membranes was performed using SEM and optical microscopy. Optical microscopy was carried out by using Leica DM LM microscope. SEM analysis was performed by a Carl Zeiss EVO^®^ 40 EP apparatus coupled with EDX at 15 kV. For the cross-section images, the samples were immerged in liquid nitrogen and cryo-fractured. Prior to the observation, samples were metallized with palladium.

#### 2.6.6. Tensile Tests

The tensile measurements were carried out with a universal tensile machine (Instron 5543) with the 500 N-load cell, according to ISO 527-A. All tests were carried out at the crosshead speed of 1 mm/min at room temperature (24 ± 1 °C) and hygrometry (48 ± 2% of relative humidity). For each sample, a minimum of seven specimens were tested and the mean values of Young’s modulus (*E*) and elongation at break (*ε*) were calculated.

#### 2.6.7. Bubble Point Test

The details of this method are given in [25]. Briefly, the impregnated Matrimid^®^ membrane was mounted in a circular housing within a stainless-steel filter holder (Millipore, XX3001200). The upstream part of the filter holder was connected to inert gas (N_2_) by a precision pressure gauge ranging from 0 to 0.1 bar. The downstream part of the filter holder was connected to a pipe immersed in water at its lower end. At the beginning of the measurement, there was no difference between the upstream and downstream pressures (i.e., the transmembrane pressure). Then, the upstream pressure was slowly and gradually increased by a step of 0.002 bar every 2 min. Until the pressure difference over the impregnated membrane reached the capillary pressure of the pores, the PIL acted as a barrier and no flow was permitted through the membrane. However, when the pressure exceeded this limit, the first gas bubbles appeared in the water at the downstream part. This pressure value corresponds to the bubble point pressure Δ*P*. Each measurement was repeated at least three times for reproducibility.

#### 2.6.8. Ionic Conductivity

The ionic conductivity was measured by electrochemical impedance spectroscopy using an impedance spectrometer (M2 Materials Mates 7620 impedance analyzer) with four electrodes cell (surface conductivity) in the frequency range from 0.1 to 5 MHz and in the temperature range between 25 °C and 150 °C. The recorded impedance data were presented in the Nyquist plane plot. The sample resistance was determined from the circle response on the real axis Z’ of the complex impedance plot.

## 3. Results and Discussion

### 3.1. Protic Imidazolium-Based Ionic Liquids—VImTf, AImTf and MimTf

It has been shown that using nonaqueous proton carriers, instead of water, is a simple and effective method of obtaining polymer electrolyte membranes with a high proton conductivity at temperatures higher than 100 °C [18]. Therefore, PILs have attracted much attention as proton carriers, as they can be used at temperatures above 100 °C under anhydrous conditions without conductivity decreases due to their negligible volatility and excellent thermal stability. However, PILs dispersed in a polymer matrix are usually water-soluble and, thus, they can be easily taken away from the membrane by the water generated at the cathode of the fuel cell [1,16]. In the literature, a variety of methods of PILs immobilization in the membrane has been proposed [25,29]. These methods may be divided into two groups: (i) PIL absorption within a polymer matrix, and (ii) PIL grafting to the polymer matrix by covalent bonding. In the first case (i.e., PIL impregnation) the PIL can easily and freely move within the polymer matrix, thus causing different secondary reactions and the conductivity decrease owing to the PIL release [29]. In the second case (i.e., the PIL grafting), a reduction in proton conductivity may be observed because of decreased ion mobility [22]. Therefore, the combination of PILs and their polymer analogs within the polymer matrix may open new prospects for fuel-cell application. The benefit, in this case, is the fact that the ionic interactions between the PIL and its polymer analog can provide structural reinforcement of the polymer matrix, and also limit the PIL’s migration. The presence of the PIL polymer analog can provide additional proton-hopping sites, thus resulting in an increase in ion conductivity and long-term conductive stability. Taking all the aforementioned into account, PILs containing polymerizable functional groups, i.e., vinyl (VImTf), allyl (AImTf) or methacrylate (MImTf), were synthesized by the direct neutralization reaction of the Brønsted acid and base (Scheme 1). It should be mentioned that, at room temperature (25 °C), AImTf and MImTf are viscous liquids, whereas VImTf is solid.

**Figure 1:**
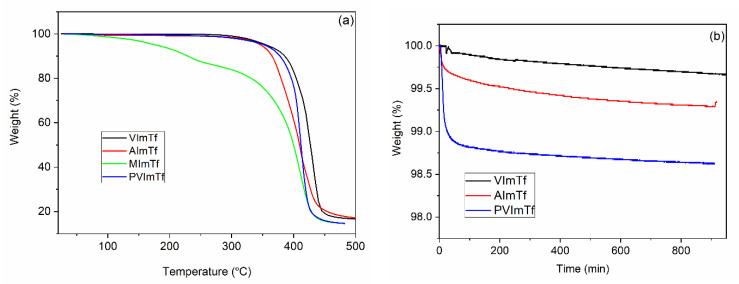
(**a**) TGA curves and (**b**) TGA isotherms at 130 °C for synthesized PILs.

The PILs’ chemical structures were confirmed by NMR analysis (see Section 2.2) and some physical–chemical parameters of the synthesized PILs (namely, different thermal transitions (i.e., melting *T*_m_, glass transition *T*_g_ and crystallization *T*_c_), viscosity η and ionic conductivity σ) are gathered in Table 1. The obtained TGA curves are shown in Figure 1a. As one can see, the temperature of 5% weight loss (*T*_5%_) of VImTf and AImTf is close to 350 °C (Table 1 and Figure 1a), thus meaning that these PILs reveal high thermal stability. However, MImTf showed rather low thermal stability (*T*_5%_ = 178 °C, Table 1). This result may be explained by the presence of bulk ester substituent, namely ethyl 2-methylacrylate (Scheme 1) that degrades at a lower temperature (Figure 1a). In order to study the long-term thermal stability of synthesized PILs, the measurements in the isothermal static mode as a function of time were carried out at 130 °C (Figure 1b). As one can see, weight loss of less than 1.5% was observed for VImTf and AImTf, thus confirming the high thermal stability of the PIL.

The obtained results of the PIL viscosity (Table 1) are in good agreement with the literature [30,31]. It was found that the decrease of the tail length of alkyl imidazolium chain caused the increasing viscosity; in fact, the viscosity value of AImTf is lower than that of VImTf—12.6 mPa·s and 17.4 mPa·s at 80 °C, respectively (Table 1). This result can be explained by the loss of flexibility of the alkyl substituent, which may create friction through interactions with neighbouring ions during rotation. However, the accurate study of the relationship between the PIL molecular structure and its viscosity is rather complex because of the presence of hydrogen bonding and other intermolecular forces [30].

**Figure 2:**
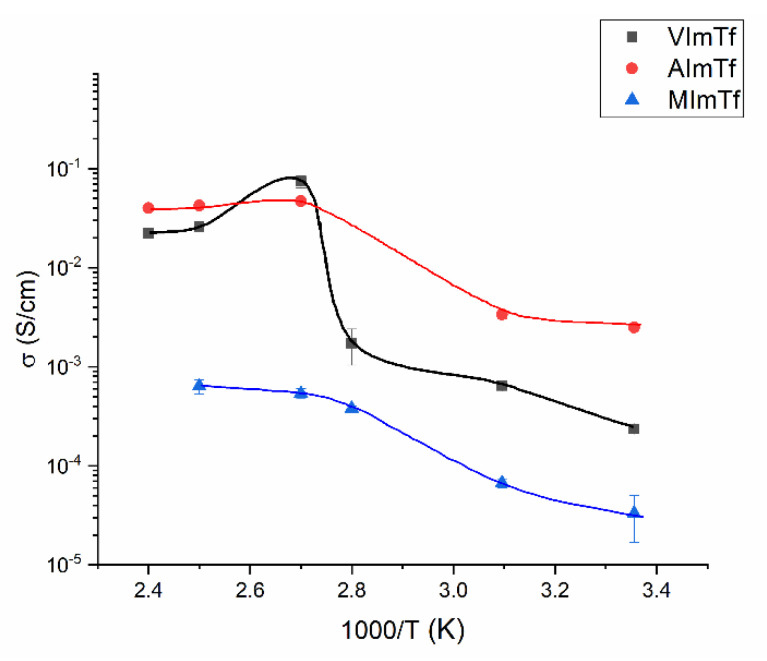
Ionic conductivity of synthesized PILs as a function of the reciprocal temperature.

The ionic conductivity of PILs is an important parameter for their potential application in different electrochemical devices. The ionic conductivity depends on the number of the carrier ions, as well as on the degree of their mobility. That is why the ionic conductivity of synthesized PILs was measured over a wide temperature range, i.e., between 25 °C and 150 °C. The influence of alkylimidazolium chain length (alkyl = vinyl for VImTf, allyl for AImTf, or ethyl methacrylate for MImTf) on the temperature dependence of the ionic conductivity for the obtained PILs can be seen in Figure 2. It is known that the value of ionic conductivity σ of individual ILs strongly depends on their structural modifications [29]. All three PILs revealed a conductivity increase with rising temperature (Figure 2). This is due to the PIL viscosity decreasing and the PIL mobility enhancing with increasing temperature, which promotes the formation of interconnected anhydrous proton transfer channels. For example, compare the viscosity of AImTf at 25 °C and 80 °C (i.e., 62.0 mPa·s and 12.6 mPa·s, respectively, Table 1). The allyl-containing ionic liquid (AImTf) shows the highest conductivity over almost the entire studied temperature range (Figure 2 and Table 1). As can be seen from the obtained data, the PIL with an ethyl methacrylate chain (MImTf) had the lowest conductivity values at any temperature. It was found that the increase of the tail length of ionic liquids or the presence of side groups grafted to the IL tail reduced the ionic conductivity [29]. For example, the IL with a methacrylate functional group showed a lower conductivity value (i.e., 0.080 mS/cm at 25 °C) than the IL with an acrylate group (0.264 mS/cm at 25 °C) at the same alkyl chain length. In the case of VImTf, the change of the conductivity slope may be observed as a function of temperature (Figure 2). This result can be explained by the fact that this PIL is solid at room temperature, as its melting temperature is 77 °C (Table 1). Thus, a significant conductivity increase was observed for this PIL at the moment of its state change (i.e., from solid to melt) due to the ion mobility increase.

To characterize the proton transport mechanism in the synthesized PILs, the conductivity activation energy was determined. The relationship of the temperature with the proton conductivity follows the Arrhenius equation:

(2) $$\sigma =\sigma_{0}exp\;\left(-\frac{E_{a}}{RT}\right),$$

where σ is the proton conductivity, $\sigma_{0}$ is the pre-exponential factor, *E*_a_ is the activation energy of the proton conduction, *R* is the gas constant and *T* is the temperature. It can be seen from Table 1 that the determined values of *E*_a_ range from ~12 kJ/mol to ~20 kJ/mol. Thus, according to the literature [26], the conduction mechanism follows the Grotthuss mechanism (*E*_a_ ranging from 14 kJ/mol to 40 kJ/mol), also known as proton-jumping through the hydrogen-bond network.

Since the polymer electrolyte membrane stays at elevated temperatures for a long time (sometimes up to some months) during the fuel cell’s operation, long-term performance is a very important parameter [32,33]. Therefore, the thermal stability of the conductivity of the synthesized PILs was evaluated as a function of time at 130 °C (Figure S1) as well as a function of the temperature in cyclic heating/cooling mode (Figure S2). One can see that no significant decline in conductivity was observed for all PILs within 600 min at 130 °C, thus testifying that the PIL conductivity kept stable during the stability test, indicating that PILs had the satisfied stability at this temperature. In addition, during cyclic heating/cooling measurements, no significant difference between different cycles was observed for VImTf and AImTf (Figure S1a,b, respectively). The conductivity changes were observed close to the VImTf melting temperature regardless of cycle (i.e., heating or cooling). However, a subsequent conductivity loss was observed for MImTf in the temperature cycling, over almost the entire tested temperature range (Figure S2c). Therefore, taking this into account (namely, the lowest thermal stability and conductivity loss), MImTf was not further studied.

### 3.2. Polymer PILs

As it was already mentioned, PILs can be rather easily released from the polymer matrix. Thus, to prevent this process, PIL should be entrapped inside the polymer. To do that, the study of the polymerization of the synthesized PILs was carried out.

**Scheme 2:**
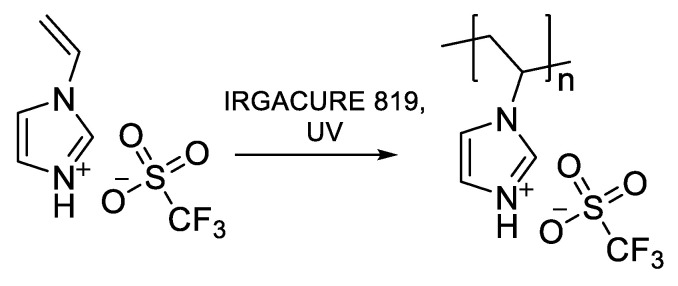
VImTf polymerization reaction.

In case of VImTf, Irgacure^®^ 819 was chosen as a standard type I photoinitiator for the free radical polymerization reaction (Scheme 2).

**Figure 3:**
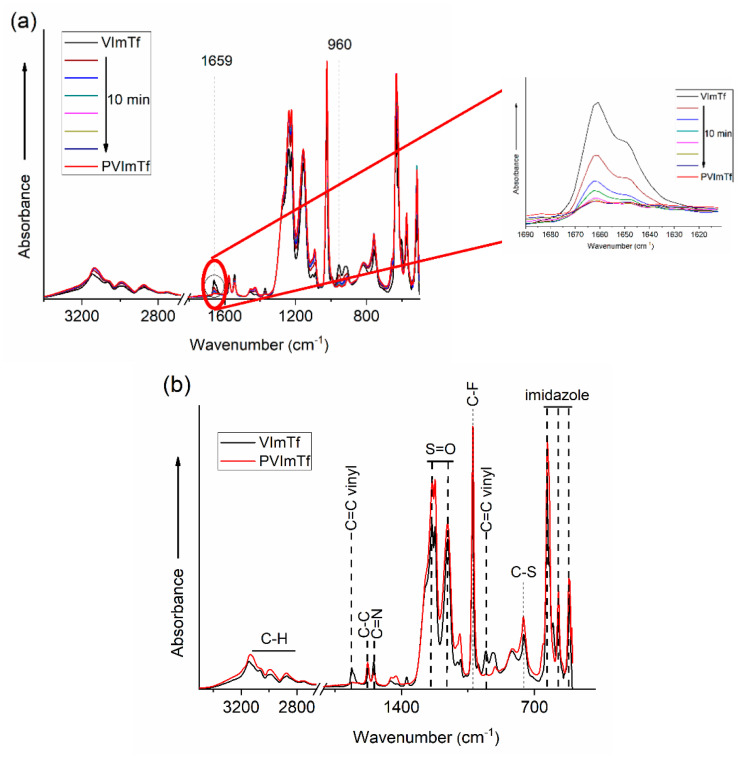
FTIR spectra of (**a**) VImTf and PVImTf and of (**b**) VImTf as a function of different polymerization time.

The photopolymerization was followed at 25 °C by FTIR-ATR spectroscopy analysis (Figure 3). As one can see, before the polymerization the spectrum of VImTf was characterized by the absorbance at 3100–2940 cm^−1^ attributed to C-H stretching vibrations of the imidazolium ring, by the bands at 1659 cm^−1^ and 960 cm^−1^ attributed to C=C vinyl stretching, by the vibrations at 1573 cm^−1^ and 1544 cm^−1^ corresponding to C–C and C=N ring stretching, respectively [32,33]. The observed vibrations at 624–514 cm^−1^ correspond to the vibrations within the imidazole ring. S=O stretching vibrations can be seen at 1240 cm^−1^ and 1156 cm^−1^. The bands at 1019 cm^−1^ and at 757 cm^−1^ are attributed to C-F stretching and C-S stretching of anion (i.e., trifluoromethane sulfonate), respectively.

During the photopolymerization, the FTIR spectrum of VImTf was measured regularly in order to study the polymerization kinetics. As one can see from Figure 3b, the bands at 1659 cm^−1^ and 960 cm^−1^, assigned to C=C vinyl stretching, disappeared and an increase of absorption bands at 3100–2940 cm^−1^, 1448 cm^−1^ and at 1432 cm^−1^ related to the C–H stretching, C–C stretching and C–H stretching of the alkane backbone, respectively, was noted, thus confirming the PIL’s polymerization. This result may be explained by the system’s reorganization and by the molecular interaction caused by the PIL’s polymerization [33].

In order to determine the conversion degree, the presence of chemical bonds with isolated absorption bands, which can serve as the internal standard, is required. However, sometimes it is rather difficult to find such absorption bands due to the bands’ overlapping. Therefore, several absorption bands may be chosen as the internal standard for a more precise determination of the conversion degree. Thus, in our case absorption bands at 1573, 1544 and 1024 cm^−1^ were chosen. The conversion degree *%C* was calculated using the integral peak intensity of the absorption bands as follows:

(3) $$\%C=\frac{Abs\left(vinyl\right)\;/\;Abs\left(internal\;standard\right)polymer}{Abs\left(vinyl\right)\;/\;Abs\left(internal\;standard\right)monomer}\;\cdot 100\%$$

and was determined to be 76.2 ± 3.0%.

As for the AImTf polymerization, several methods were tested, namely radical photopolymerization in the presence of Irgacure^®^ 819 (type I photoinitiator) or benzophenone (type II photoinitiator), and thermal radical polymerization at 70 °C, using the azobisisobutyronitrile initiator and thiol derivatives (C_10_H_22_S, C_6_H_14_S and C_9_H_22_O_3_SiS). Additionally, cationic polymerization, using a titanium tetrachloride initiator in dichloromethane at −10 °C, was also tried. However, no polymer was obtained in any case, independent of initiator concentration. It is known that allyl monomers polymerize very slowly, as allyl radicals are much more stable than corresponding vinyl radicals [34,35].

Taking these results into account, only PVImTf was further analyzed. The results of the performed thermal analysis of PVImTf show that its thermal stability was comparable with the thermal stability of VImTf, i.e., the temperature of 5% weight loss is 353 °C for PVImTf and 355 °C for VImTf (Table 1 and Figure 1). However, PVImTf did not reveal any conductivity in the studied temperature range (i.e., from 30 °C to 150 °C) despite a rather high ionic conductivity of VImTf, i.e., before the polymerization (Table 1). Such a result is rather expected, as in the case of polymer ionic liquids the decrease and/or loss of the ion segmental motion and ion binding through covalent bonds lead to a reduction of the thermal motion of the ion unit [22].

### 3.3. Composite Matrimid^®^/PIL Membrane

The obtained PILs (i.e., VImTf, AImTf and PVImTf) were used for the elaboration of SILMs based on Matrimid^®^. The polyimide membrane was chosen due to its proven versatility and commercial-scale production as well as its high glass transition (338 °C) and degradation (465 °C) temperature. SILMs were obtained by the direct immersion of the porous Matrimid^®^ membrane into the obtained PILs. The amount of immobilized PIL is listed in Table 2.

It is shown that the membrane impregnation content *τ* depends on the chemical structure and viscosity of the PILs, as well as on the impregnation conditions. As it can be seen, among all tested PILs (i.e., VImTf, AImTf and PVImTf), the Matrimid^®^/VImTf membrane was characterized by the highest *τ* value (210%, Table 2). In addition, the Matrimid^®^/PVImTf membrane showed the lowest *τ* value (146%, Table 2). It should be noted that the impregnation content for the Matrimid^®^/PVImTf membrane can be increased up to ~ 230% through increasing the PVImTf concentration in the impregnation solution (to higher than 67%). However, in this case, the obtained membrane’s mechanical properties significantly worsened and the membrane became very brittle. Thus, the maximum level of impregnation, at which the membrane’s mechanical stability was maintained, was fixed at 146%.

One can also note, from Table 2, that the Matrimid^®^/PVImTf membrane does not reveal any conductivity, at neither 50 °C nor at 100 °C. This result may be explained by a significant decrease in the proton mobility in the case of polymerized ionic liquids [22,23]. Therefore, in order to increase membrane proton conductivity and to prevent the PIL release, a three-component membrane (Matrimid^®^/VImTf/PVImTf) was prepared by the additional soaking of the Matrimid^®^/PVImTf membrane in VImTf. The impregnation value *τ* in this case was 276%, which is ~1.3 times higher than the value obtained for the Matrimid^®^/VImTf membrane. A significant increase of the *τ* value for the Matrimid^®^/VImTf/PVImTf membrane can be explained by ionic interactions between VImTf and PVImTf.

**Figure 4:**
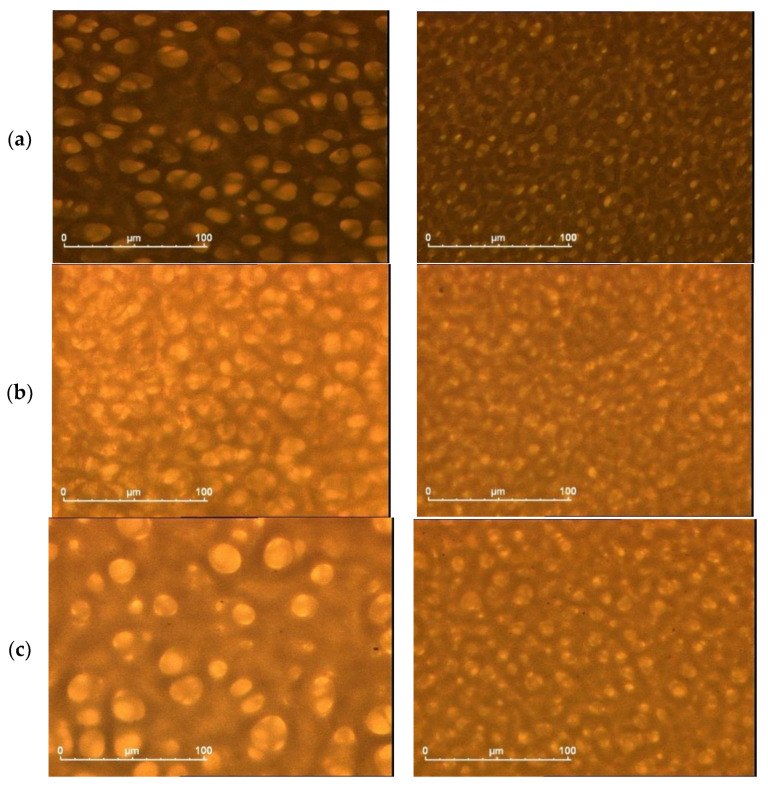
Optical images of the air- (left) and glass- (right) contacting surfaces of (**a**) Matrimid^®^, (**b**) Matrimid^®^/VImTf and (**c**) Matrimid^®^/AImTf.

The morphology of the elaborated SILMs was investigated by optical microscopy and SEM. Optical microscopy images were obtained for each membrane from both air- and glass-contacting surfaces (Figure 4). As expected, the optical images reveal that the Matrimid^®^ membrane possesses an open porosity structure, with large pores on the air-contacting surface (Figure 4a, left) and smaller pores on the glass-contacting surface (Figure 4a, right). This structure was preserved across all SILMs. In case of SILMs, pore boundaries are slightly blurred, especially on the glass-contacting surface of the membrane (Figure 4b,c, right). This fact is due to the pores filling with PILs.

**Figure 5:**
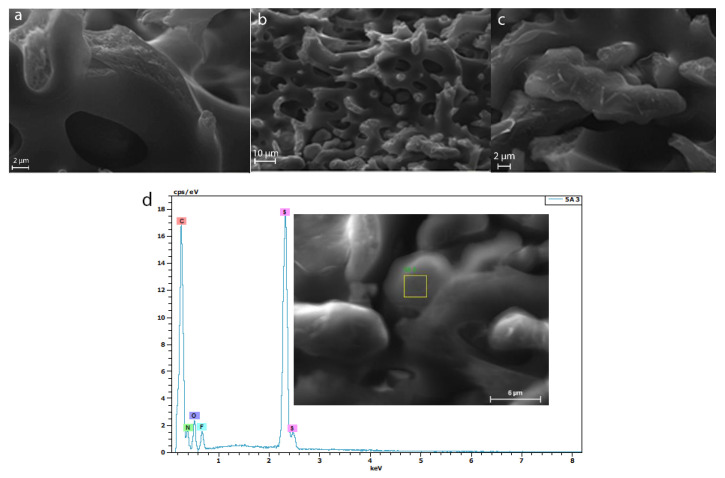
SEM images of (**a**) Matrimid^®^, (**b**,**c**) Matrimid^®^/VImTf membranes and (**d**) EDX spectrum of Matrimid^®^/VImTf membrane.

The optical microscopy data were in good agreement with SEM and EDX analyses. The Matrimid^®^ membrane is characterized by a spongy, interconnected and relatively symmetrical structure with primary (pore size between 10 and 15 µm) and secondary (pore size about 0.5 µm) porosity (Figure 5a) [24].

The SEM analysis confirmed that the porous and homogeneous structure of the pure Matrimid^®^ membrane was influenced by the PILs’ presence in the polymer matrix. The incorporation of PILs resulted in the filling of membrane pores, which was also the case in the presence of PIL-rich and Matrimid^®^-rich domains (Figure 5b,c). The performed EDX analysis also confirmed the presence of VImTf in Matrimid^®^ pores (Figure 5d). It should be mentioned that the SEM and EDX analyses were only possible in the case of solid VImTf. In the case of liquid AimTf, the PIL was removed from membrane during the vacuum step.

**Figure 6:**
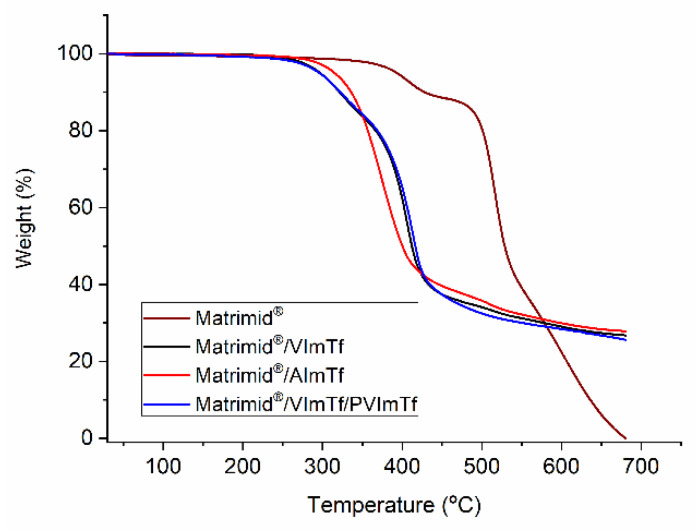
Thermogravimetrical curves of pure Matrimid^®^ and elaborated SILMs.

The membrane thermal stability was evaluated by TGA (Figure 6). The temperature values corresponding to the 5% weight loss *T_5%_* are gathered in Table 2. As one can see from the obtained results, the *T*_5%_ values are dependent on the PIL type. Moreover, a decrease in the thermal stability of SILM, in comparison with the pure PIL and with the pure Matrimid^®^ membranes, was observed (Table 1 and Table 2). The decrease of the SILM thermal stability may be explained by the catalysis reaction of the thermal degradation of polymer chains with PILs. It is known that some ILs are characterized by Brønsted or Lewis acidity, as well as by superacid properties, which are controlled and may vary over a wide range [3,20]. In its turn, the Lewis and Brønsted acids are used as catalysts in the reactions of cracking of organic compounds, isomerization, polymerization, dehydration, etc. [17,21,36]. However, the SILM thermal stability (more than 200 °C) is high enough for fuel-cell application.

**Figure 7:**
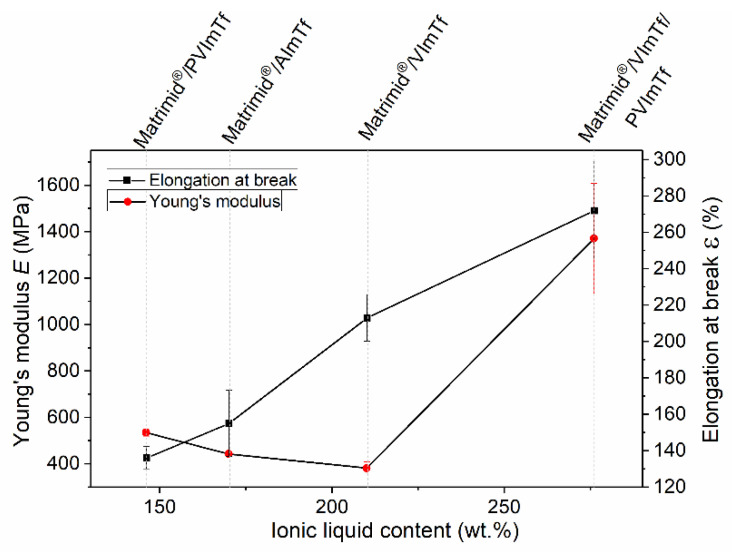
Mechanical properties of the elaborated SILMs.

The membrane mechanical properties were analyzed in terms of their *E* and *ε* values, using the standard tensile test. The obtained values of *E* and *ε* are listed in Table 2 and shown in Figure 7. It should be mentioned that whatever the PIL used is, the *E* and *ε* values are significantly higher as compared with the pure Matrimid^®^ membrane (Table 2). The membrane mechanical properties depend on the PIL nature, and on its content, i.e., an increase of the *ε* value and a decrease of the Young’s modulus value are observed with the rise of the PIL content.

In case of the three-component Matrimid^®^/VImTf/PVImTf membrane, a significant rising of both *ε* and *E* values is noted (Figure 7). This result may be explained by the fact that this membrane contained a large amount of crystalline VImTf and PVImTf, which act as mechanical amplifiers of the polymer chains and make the membrane stiffer (i.e., higher Young’s modulus values) at the same time.

In order to check the stability of the SILMs (i.e., PILs’ retention capacities) the bubble-point method was used. The obtained bubble-point pressure values ∆*P* are gathered in Table 2, thus allowing us to compare membrane stabilities. As one can see from the obtained results, the bubble-point pressure was highly dependent of the PIL used and increased in the following order:

PVImTf < AImTf < VImTf/PVImTf < VImTf.

The SILM elaborated with VImTf showed the highest stability towards an increased pressure on one of its faces, due to the fact that this PIL is solid at room temperature and, thus, difficult to remove from the membrane pores. As to the other SILMs, they revealed comparable stability under the experimental conditions.

**Figure 8:**
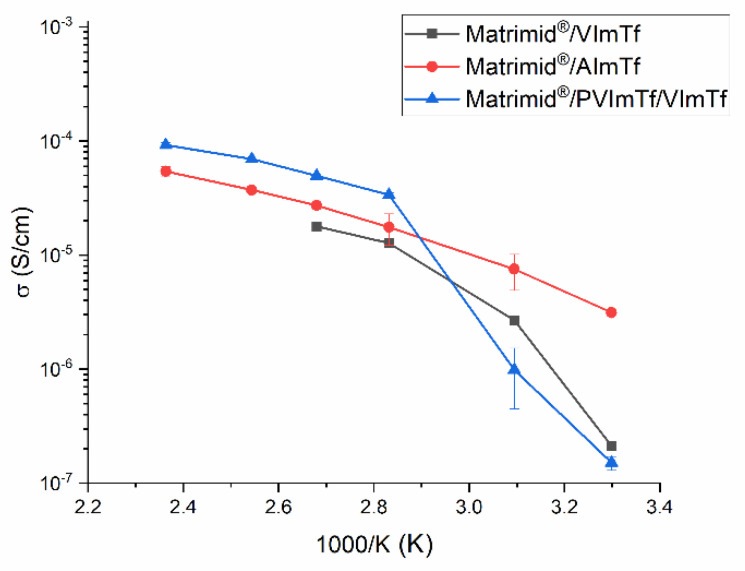
SILMs’ ionic conductivity as a function of the reciprocal temperature.

As elaborated SILMs are designed to be used as a polymer membrane in fuel cells, their membrane ionic conductivity properties are essential. Figure 8 shows the measured conductivity values of the SILMs over a wide temperature range. Also, the conductivity values obtained at 50 °C and 100 °C are listed in Table 2.

As expected, the SILMs’ conductivity was lower than that obtained for the pure PILs over the entire temperature range, owing to the reduced PIL mobility within the Matrimid^®^ membrane (compare Figure 2 and Figure 8, and Table 1 and Table 2). As a consequence, the calculated values of the activation energy *E*_a_ for the elaborated SILMs (23–26 kJ/mol) are higher than those determined for the pure PILs (12–13 kJ/mol).

The ionic conductivity of the Matrimid^®^/VImTf and Matrimid^®^/VImTf/PVImTf membranes was about 10^−3^ mS/cm at 30 °C, which is one order of magnitude lower than that of the Matrimid^®^/AImTf membrane. This result is certainly due to the fact that VImTf is solid at room temperature, so its ion mobility is strongly reduced at this temperature. However, the ionic conductivity of Matrimid^®^/VImTf and Matrimid^®^/VImTf/PVImTf increases significantly up to 10^−2^ mS/cm after the temperature rising to 100 °C. Besides, it should be mentioned that at 100 °C the membrane was measured under anhydrous conditions. Moreover, the three-component Matrimid^®^/VImTf/PImTf membrane revealed a slightly higher ionic conductivity in comparison with the Matrimid^®^/AImTf membrane at T ≥ 100 °C. This difference can be explained by the higher impregnation content *τ* value, in case of the Matrimid^®^/VImTf/PVImTf membrane—170% and 276% for Matrimid^®^/AImTf and Matrimid^®^/VImTf/PVImTf, respectively (Table 2). In any case, all the studied SILMs showed good electrochemical stability, as determined by ionic conductivity measurements in a cyclic mode from 30 °C to 140 °C for more than 9000 min. It was found that the Matrimid^®^/VImTf and Matrimid^®^/AImTf membranes had lost less than 1% of their weight after measurement, and the Matrimid^®^/VImTf/PVImTf membrane had lost ~2% of its weight. Such results are rather promising for their application as membranes for fuel cells at high temperatures.

## 4. Conclusions

PILs containing polymerizable vinyl, allyl, or methacrylate groups and a polymer PIL were synthesized and their physical–chemical properties, as well as their ionic conductivities, were determined. It was demonstrated that the PILs’ ionic conductivity strongly depended on their alkylimidazolium chains’ length. The measured conductivity values of VImTf_,_ AImTf and MImTf were 26.0, 42.2 and 0.64 mS/cm at 130 °C, respectively. The lower conductivity of MImTf is explained by its increasing tail length and by its low thermal stability.

PILs with vinyl and allyl functional groups were photopolymerized using different photoinitiators. However, in the case of AImTf, no polymer was obtained whatever photoinitiator was used. Such a result may be explained by the higher stability of allyl monomers as compared with the vinyl ones. The synthesized VImTf, AImTf and PVImTf PILs possessed good thermal (*T*_5%_ > 300 °C) and electrochemical stability in dynamic and static experimental modes, as no significant conductivity decline was observed over 600 min at 130 °C.

SILMs based on Matrimid^®^ were prepared by the direct immersion in VImTf, AImTf and PVImTf. All obtained membranes show excellent thermal stability (*T*_5%_ ~ 300 °C) and mechanical properties (*ε* > 130%) as well as good conductivity. It is found that the using both a PIL and its polymer analog in the same membrane (i.e., Matrimid^®^/VImTf/PVImTf) leads to SLIMs with higher conductivities (i.e., 0.05 mS/cm) as compared with membranes with only one component (i.e., PIL or polymer PIL). This result clearly reveals the interest of composite membranes, which consist of a microporous thermally stable polymer, used as a support, and impregnated by a mixture of PILs and polymer PILs that bring high ionic conductivity while maintaining stability at high temperature.

## Figures and Tables

**Table 1:** Physical–chemical characteristics of the synthesized PILs.

PILs	*T*_g_ (°C)	*T*_m_ (°C)	*T*_c_ (°C)	*T*_5%_ (°C)	η (mPa·s)	σ (mS/cm)		*E*_a_ (kJ/mol)
						at 50 °C	at 130 °C	
VImTf	−1.4	77.0	30.0	355	17.4 *	0.64	26.0	20.2
AImTf	-	11.7	−39.0	347	62.0 12.6 *	3.37	42.4	12.4
MImTf	−55.0	-	-	178	-	0.07	0.64	13.9
PVImTf	124.5	-	-	353	25.4 *	-	-	-

* at 80 °C. *T*_m_—melting temperature; *T*_g_—glass transition temperature; *T*_c_—crystallization temperature; *T*_5%_—temperatures of 5% weight loss; η—viscosity; σ—proton conductivity; *E*_a_—activation energy.

**Table 2:** Impregnation content, thermal and mechanical stability and ionic conductivity of elaborated SLIMs.

Membrane	*T*_5%_ (%)	Impregnation Content *τ* (wt.%)	Young’s Modulus *E* (MPa)	Elongation at Break *ε* (%)	Bubble Point Pressure Δ*P* (bar)	Ionic Conductivity σ (mS/cm)		Activation Energy *E*_a_ (kJ/mol)
						at 50 °C	at 100 °C	
Matrimid^®^	397	-	314 ± 8	35 ± 10	-	-	-	-
Matrimid^®^/VImTf	291	210 ± 16	382 ± 26	213 ± 13	>1	2.7 × 10^−3^	1.8 × 10^−2^	25
Matrimid^®^/AImTf	310	170 ± 18	443 ± 18	155 ± 10	0.09 ± 0.01	7.5 × 10^−3^	2.7 × 10^−2^	23
Matrimid^®^/PVImTf	290	146 ± 6	535 ± 16	136 ± 6	0.05 ± 0.01	-	-	-
Matrimid^®^/VImTf/PVImTf	290	276 ± 16	1371 ± 237	272 ± 27	0.85 ± 0.02	9.9 × 10^−4^	4.9 × 10^−2^	26

## Data Availability

The data are contained within the article.

## Supplementary Materials

The following are available online at https://www.mdpi.com/article/10.3390/polym13213704/s1, Figure S1: PIL conductivity as a function of time at 130 °C, Figure S2: Conductivity of synthesized PILs as a function of temperature in cyclic heating/cooling mode: (**a**) VImTf, (**b**) AImTf and (**c**) MImTf.
