# Supplementary material for: Novel Ionic Conducting Composite Membrane Based on Polymerizable Ionic Liquids
Source: Polymers (Basel). 2021 Oct 27;13(21):3704. doi: 10.3390/polym13213704 (PMC8588533; doi:10.3390/polym13213704)
Supplement: Supplementary file 1 [file polymers-13-03704-s001.zip › polymers-1427444-supplementary.pdf]

Supporting Information

Novel ionic conducting composite membrane based on polymerizable ionic liquids

Ya.L. Kobzar[1], G. Azzouz[1], H. Albadri[2], J. Levillain[2], I. Dez[2], A.-C. Gaumont[2], L. Lecamp[1], C. Chappey[1], S. Marais[1], K. Fatyeyeva[1]*

[1]*Normandie Univ, UNIROUEN, INSA Rouen, CNRS, Polymerès Biopolymères Surfaces, 76000 Rouen, France*

[2]*Normandie Univ, ENSICAEN, UNICAEN, CNRS, Laboratoire de Chimie Moléculaire et Thioorganique, 14000 Caen, France*

*Corresponding author: kateryna.fatyeyeva@univ-rouen.fr

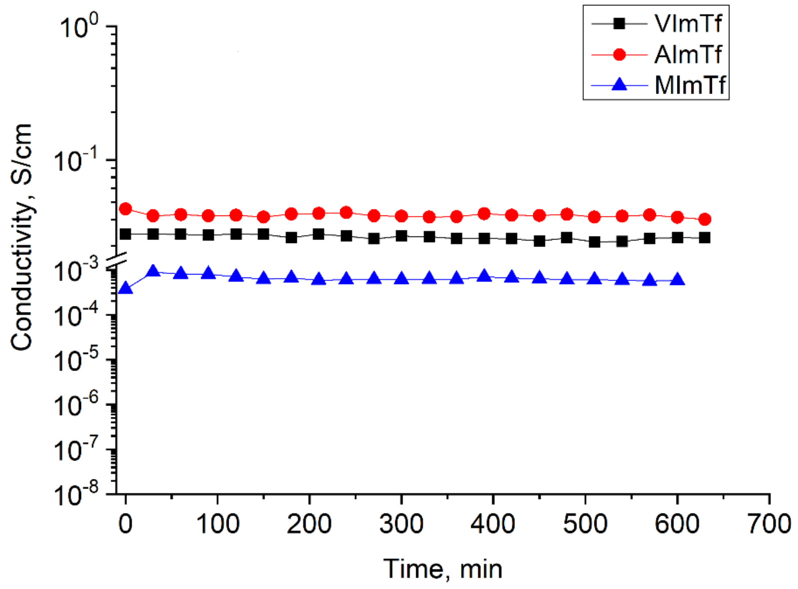

Figure S1. PIL conductivity as a function of time at 130 °C.

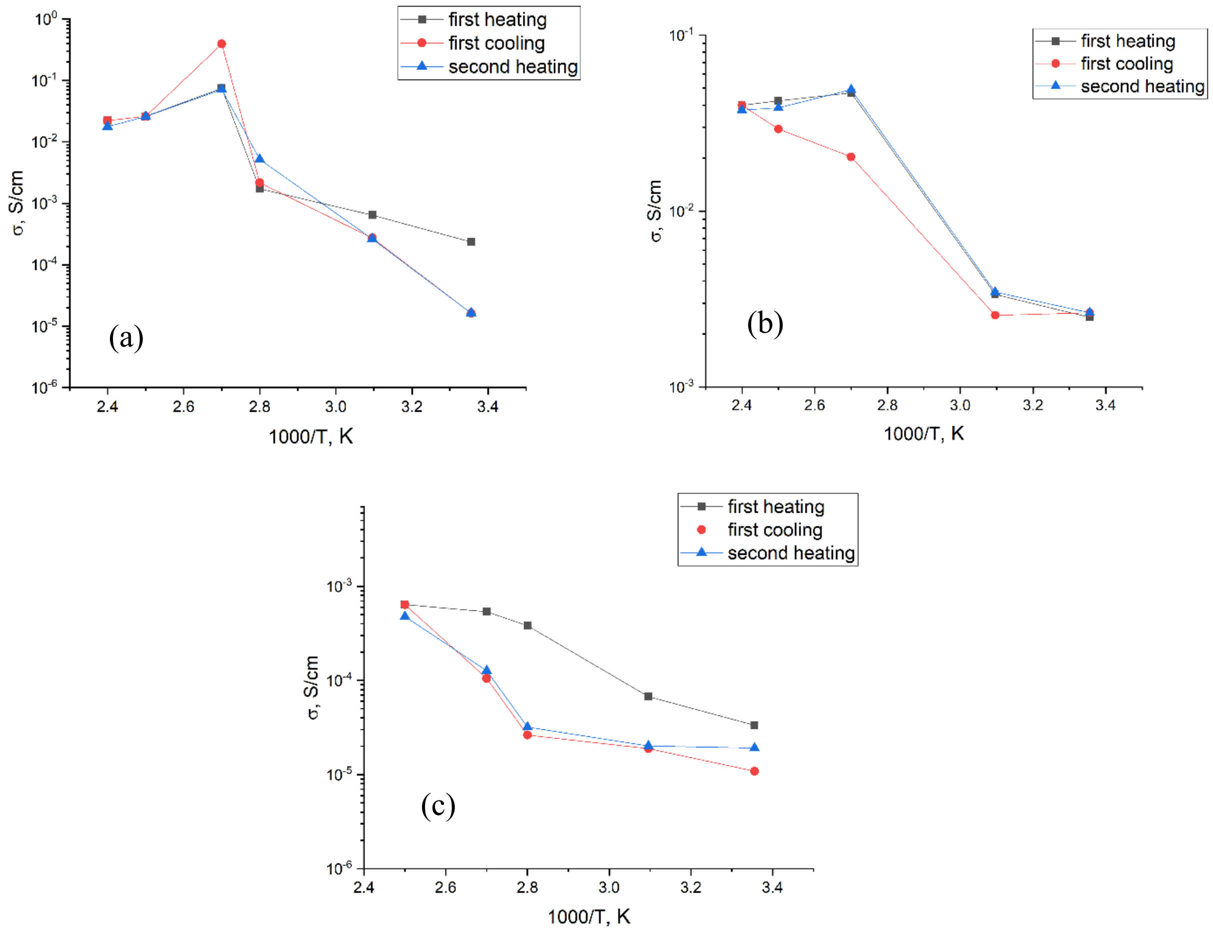

Figure S2. Conductivity of synthesized PILs as a function of temperature in cyclic heating/cooling mode: (*a*) **VImTf**, (*b*) **AImTf** and (*c*) **MImTf**.
